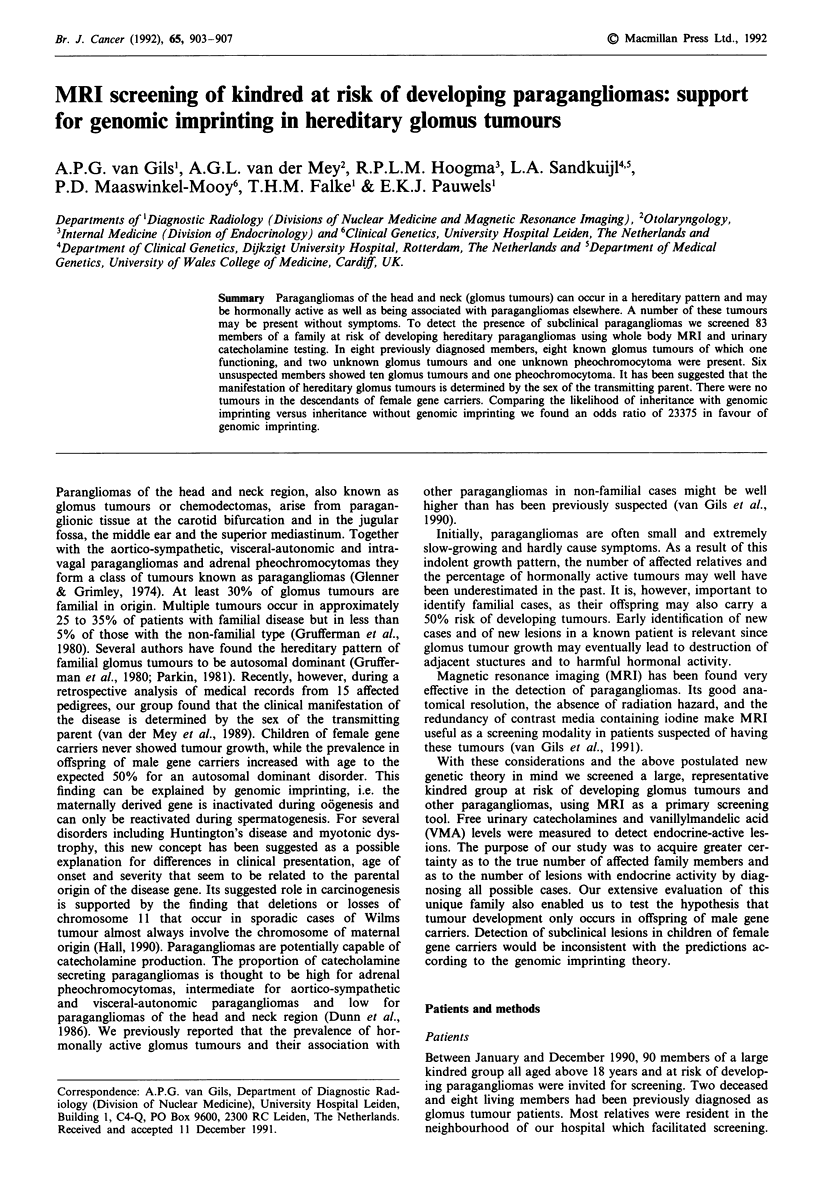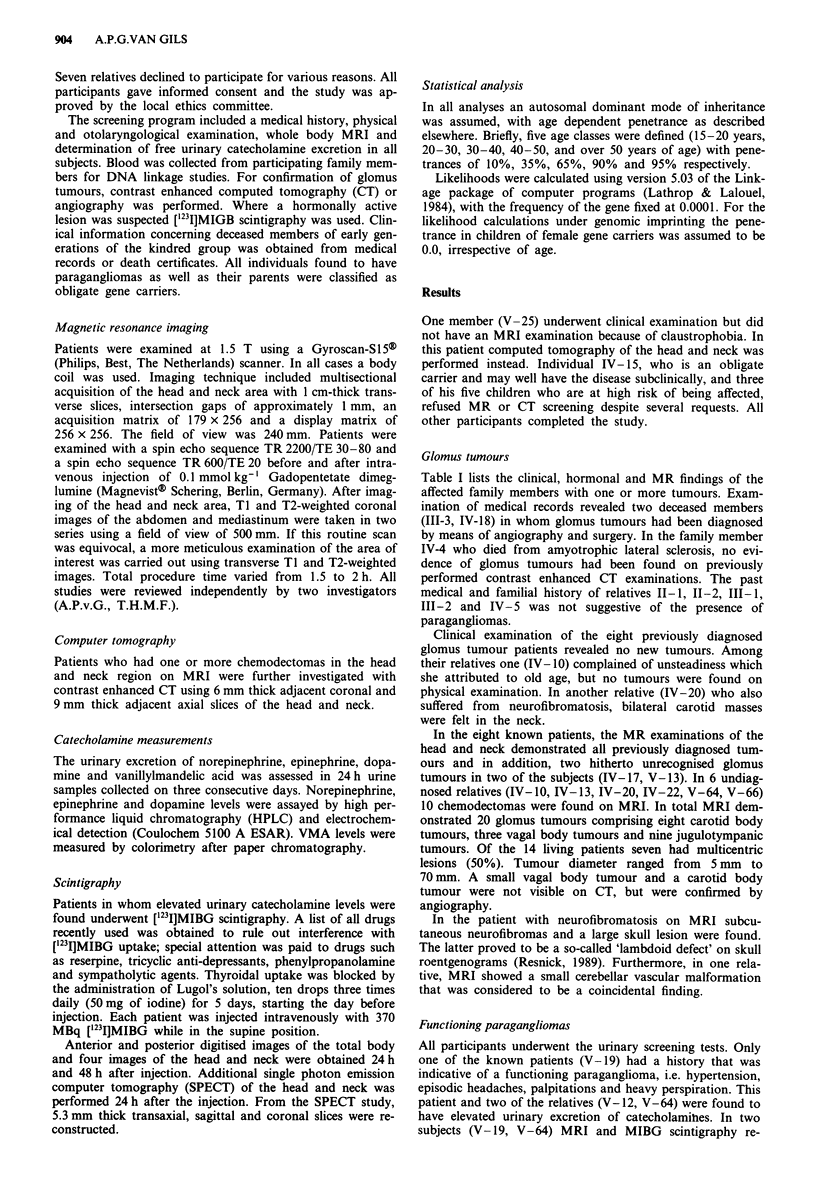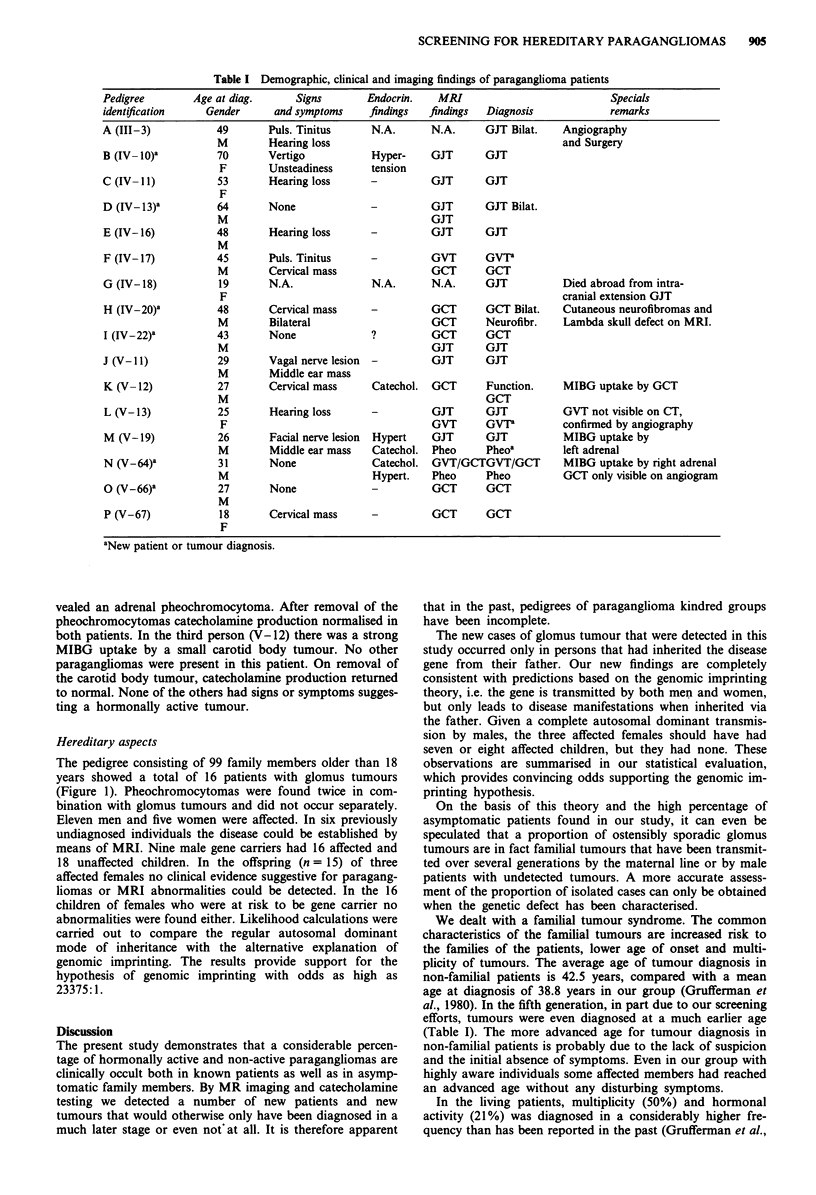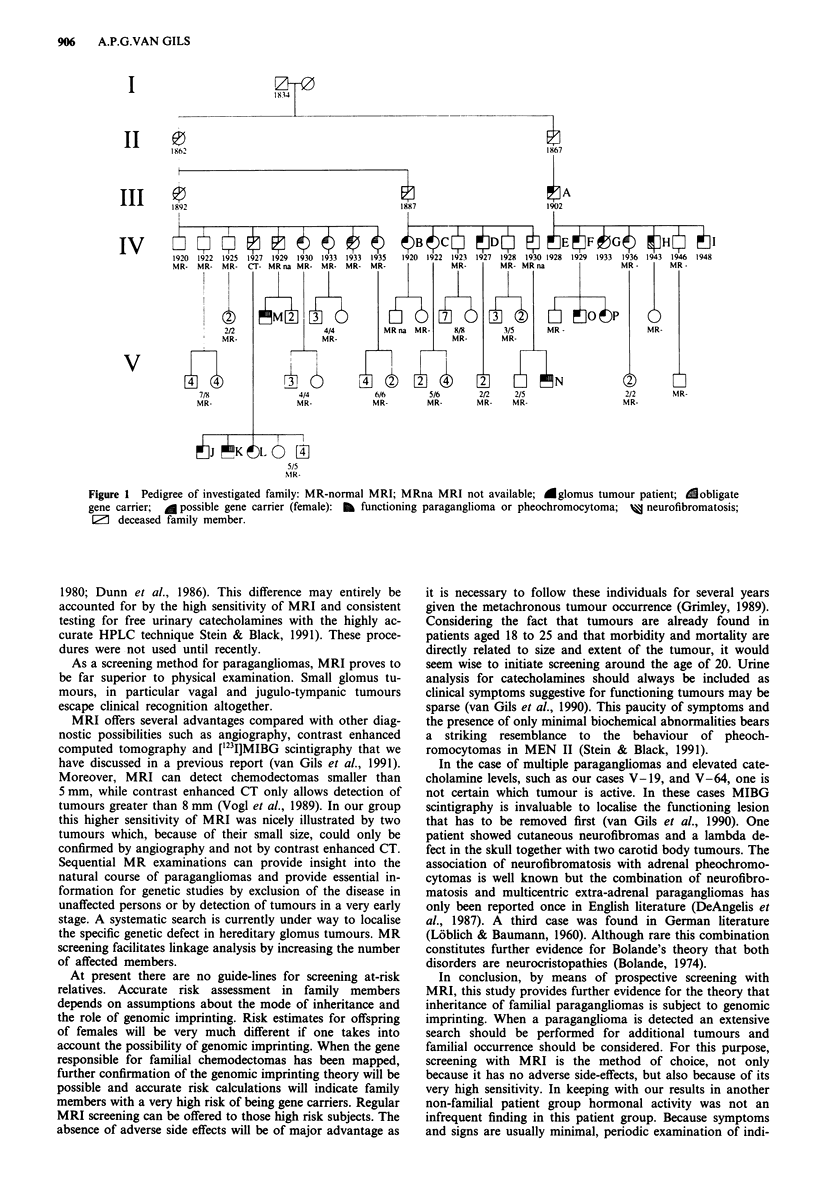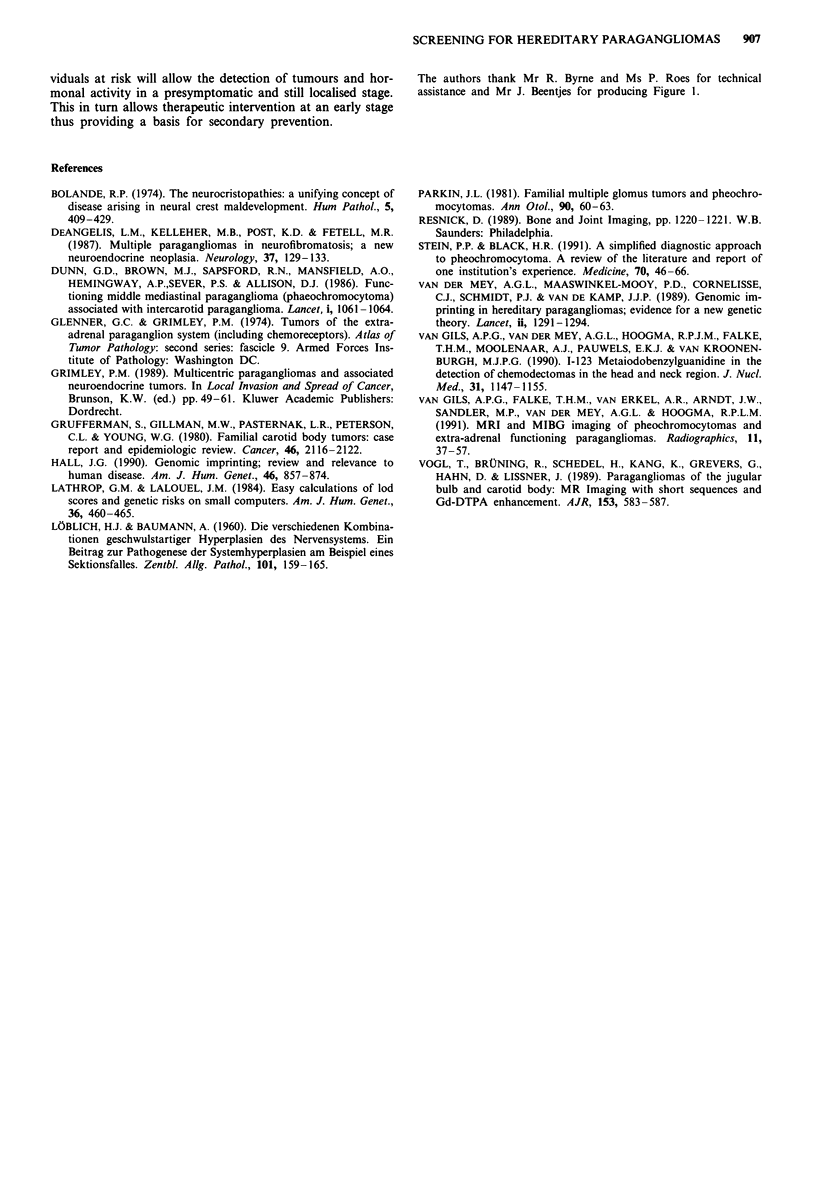# MRI screening of kindred at risk of developing paragangliomas: support for genomic imprinting in hereditary glomus tumours.

**DOI:** 10.1038/bjc.1992.189

**Published:** 1992-06

**Authors:** A. P. van Gils, A. G. van der Mey, R. P. Hoogma, L. A. Sandkuijl, P. D. Maaswinkel-Mooy, T. H. Falke, E. K. Pauwels

**Affiliations:** Department of Diagnostic Radiology (Divisions of Nuclear Medicine and Magnetic Resonance Imaging), University Hospital Leiden, The Netherlands.

## Abstract

Paragangliomas of the head and neck (glomus tumours) can occur in a hereditary pattern and may be hormonally active as well as being associated with paragangliomas elsewhere. A number of these tumours may be present without symptoms. To detect the presence of subclinical paragangliomas we screened 83 members of a family at risk of developing hereditary paragangliomas using whole body MRI and urinary catecholamine testing. In eight previously diagnosed members, eight known glomus tumours of which one functioning, and two unknown glomus tumours and one unknown pheochromocytoma were present. Six unsuspected members showed ten glomus tumours and one pheochromocytoma. It has been suggested that the manifestation of hereditary glomus tumours is determined by the sex of the transmitting parent. There were no tumours in the descendants of female gene carriers. Comparing the likelihood of inheritance with genomic imprinting versus inheritance without genomic imprinting we found an odds ratio of 23375 in favour of genomic imprinting.


*Br. J. Cancer* (1992), **65**, 903–907 © Macmillan Press Ltd., 1992

### MRI screening of kindred at risk of developing paragangliomas: support for genomic imprinting in hereditary glomus tumours

A.P.G. van Gils[1], A.G.L. van der Mey[2], R.P.L.M. Hoogma[3], L.A. Sandkuijl[4,5], P.D. Maaswinkel-Mooy[6], T.H.M. Falke[1] & E.K.J. Pauwels[1]

*Departments of [1]Diagnostic Radiology (Divisions of Nuclear Medicine and Magnetic Resonance Imaging), [2]Otolaryngology, [3]Internal Medicine (Division of Endocrinology) and [6]Clinical Genetics, University Hospital Leiden, The Netherlands and [4]Department of Clinical Genetics, Dijkzigt University Hospital, Rotterdam, The Netherlands and [5]Department of Medical Genetics, University of Wales College of Medicine, Cardiff, UK.*

**Summary** Paragangliomas of the head and neck (glomus tumours) can occur in a hereditary pattern and may be hormonally active as well as being associated with paragangliomas elsewhere. A number of these tumours may be present without symptoms. To detect the presence of subclinical paragangliomas we screened 83 members of a family at risk of developing hereditary paragangliomas using whole body MRI and urinary catecholamine testing. In eight previously diagnosed members, eight known glomus tumours of which one functioning, and two unknown glomus tumours and one unknown pheochromocytoma were present. Six unsuspected members showed ten glomus tumours and one pheochromocytoma. It has been suggested that the manifestation of hereditary glomus tumours is determined by the sex of the transmitting parent. There were no tumours in the descendants of female gene carriers. Comparing the likelihood of inheritance with genomic imprinting versus inheritance without genomic imprinting we found an odds ratio of 23375 in favour of genomic imprinting.

Parangliomas of the head and neck region, also known as glomus tumours or chemodectomas, arise from paraganglionic tissue at the carotid bifurcation and in the jugular fossa, the middle ear and the superior mediastinum. Together with the aortico-sympathetic, visceral-autonomic and intravagal paragangliomas and adrenal pheochromocytomas they form a class of tumours known as paragangliomas (Glenner & Grimley, 1974). At least 30% of glomus tumours are familial in origin. Multiple tumours occur in approximately 25 to 35% of patients with familial disease but in less than 5% of those with the non-familial type (Grufferman *et al.*, 1980). Several authors have found the hereditary pattern of familial glomus tumours to be autosomal dominant (Grufferman *et al.*, 1980; Parkin, 1981). Recently, however, during a retrospective analysis of medical records from 15 affected pedigrees, our group found that the clinical manifestation of the disease is determined by the sex of the transmitting parent (van der Mey *et al.*, 1989). Children of female gene carriers never showed tumour growth, while the prevalence in offspring of male gene carriers increased with age to the expected 50% for an autosomal dominant disorder. This finding can be explained by genomic imprinting, i.e. the maternally derived gene is inactivated during oögenesis and can only be reactivated during spermatogenesis. For several disorders including Huntington's disease and myotonic dystrophy, this new concept has been suggested as a possible explanation for differences in clinical presentation, age of onset and severity that seem to be related to the parental origin of the disease gene. Its suggested role in carcinogenesis is supported by the finding that deletions or losses of chromosome 11 that occur in sporadic cases of Wilms tumour almost always involve the chromosome of maternal origin (Hall, 1990). Paragangliomas are potentially capable of catecholamine production. The proportion of catecholamine secreting paragangliomas is thought to be high for adrenal pheochromocytomas, intermediate for aortico-sympathetic and visceral-autonomic paragangliomas and low for paragangliomas of the head and neck region (Dunn *et al.*, 1986). We previously reported that the prevalence of hormonally active glomus tumours and their association with other paragangliomas in non-familial cases might be well higher than has been previously suspected (van Gils *et al.*, 1990).

Initially, paragangliomas are often small and extremely slow-growing and hardly cause symptoms. As a result of this indolent growth pattern, the number of affected relatives and the percentage of hormonally active tumours may well have been underestimated in the past. It is, however, important to identify familial cases, as their offspring may also carry a 50% risk of developing tumours. Early identification of new cases and of new lesions in a known patient is relevant since glomus tumour growth may eventually lead to destruction of adjacent stuctures and to harmful hormonal activity.

Magnetic resonance imaging (MRI) has been found very effective in the detection of paragangliomas. Its good anatomical resolution, the absence of radiation hazard, and the redundancy of contrast media containing iodine make MRI useful as a screening modality in patients suspected of having these tumours (van Gils *et al.*, 1991).

With these considerations and the above postulated new genetic theory in mind we screened a large, representative kindred group at risk of developing glomus tumours and other paragangliomas, using MRI as a primary screening tool. Free urinary catecholamines and vanillylmandelic acid (VMA) levels were measured to detect endocrine-active lesions. The purpose of our study was to acquire greater certainty as to the true number of affected family members and as to the number of lesions with endocrine activity by diagnosing all possible cases. Our extensive evaluation of this unique family also enabled us to test the hypothesis that tumour development only occurs in offspring of male gene carriers. Detection of subclinical lesions in children of female gene carriers would be inconsistent with the predictions according to the genomic imprinting theory.

### Patients and methods

### *Patients*

Between January and December 1990, 90 members of a large kindred group all aged above 18 years and at risk of developing paragangliomas were invited for screening. Two deceased and eight living members had been previously diagnosed as glomus tumour patients. Most relatives were resident in the neighbourhood of our hospital which facilitated screening.

Correspondence: A.P.G. van Gils, Department of Diagnostic Radiology (Division of Nuclear Medicine), University Hospital Leiden, Building 1, C4-Q, PO Box 9600, 2300 RC Leiden, The Netherlands.
Received and accepted 11 December 1991.

Seven relatives declined to participate for various reasons. All participants gave informed consent and the study was approved by the local ethics committee.

The screening program included a medical history, physical and otolaryngological examination, whole body MRI and determination of free urinary catecholamine excretion in all subjects. Blood was collected from participating family members for DNA linkage studies. For confirmation of glomus tumours, contrast enhanced computed tomography (CT) or angiography was performed. Where a hormonally active lesion was suspected [$^{123}$I]MIGB scintigraphy was used. Clinical information concerning deceased members of early generations of the kindred group was obtained from medical records or death certificates. All individuals found to have paragangliomas as well as their parents were classified as obligate gene carriers.

*Magnetic resonance imaging*

Patients were examined at 1.5 T using a Gyroscan-S15® (Philips, Best, The Netherlands) scanner. In all cases a body coil was used. Imaging technique included multisectional acquisition of the head and neck area with 1 cm-thick transverse slices, intersection gaps of approximately 1 mm, an acquisition matrix of 179 × 256 and a display matrix of 256 × 256. The field of view was 240 mm. Patients were examined with a spin echo sequence TR 2200/TE 30–80 and a spin echo sequence TR 600/TE 20 before and after intravenous injection of 0.1 mmol kg$^{-1}$ Gadopentetate dimeglumine (Magnevist® Schering, Berlin, Germany). After imaging of the head and neck area, T1 and T2-weighted coronal images of the abdomen and mediastinum were taken in two series using a field of view of 500 mm. If this routine scan was equivocal, a more meticulous examination of the area of interest was carried out using transverse T1 and T2-weighted images. Total procedure time varied from 1.5 to 2 h. All studies were reviewed independently by two investigators (A.P.v.G., T.H.M.F.).

*Computer tomography*

Patients who had one or more chemodectomas in the head and neck region on MRI were further investigated with contrast enhanced CT using 6 mm thick adjacent coronal and 9 mm thick adjacent axial slices of the head and neck.

*Catecholamine measurements*

The urinary excretion of norepinephrine, epinephrine, dopamine and vanillylmandelic acid was assessed in 24 h urine samples collected on three consecutive days. Norepinephrine, epinephrine and dopamine levels were assayed by high performance liquid chromatography (HPLC) and electrochemical detection (Coulochem 5100 A ESAR). VMA levels were measured by colorimetry after paper chromatography.

*Scintigraphy*

Patients in whom elevated urinary catecholamine levels were found underwent [$^{123}$I]MIBG scintigraphy. A list of all drugs recently used was obtained to rule out interference with [$^{123}$I]MIBG uptake; special attention was paid to drugs such as reserpine, tricyclic anti-depressants, phenylpropanolamine and sympatholytic agents. Thyroidal uptake was blocked by the administration of Lugol's solution, ten drops three times daily (50 mg of iodine) for 5 days, starting the day before injection. Each patient was injected intravenously with 370 MBq [$^{123}$I]MIBG while in the supine position.

Anterior and posterior digitised images of the total body and four images of the head and neck were obtained 24 h and 48 h after injection. Additional single photon emission computer tomography (SPECT) of the head and neck was performed 24 h after the injection. From the SPECT study, 5.3 mm thick transaxial, sagittal and coronal slices were reconstructed.

*Statistical analysis*

In all analyses an autosomal dominant mode of inheritance was assumed, with age dependent penetrance as described elsewhere. Briefly, five age classes were defined (15–20 years, 20–30, 30–40, 40–50, and over 50 years of age) with penetrances of 10%, 35%, 65%, 90% and 95% respectively.

Likelihoods were calculated using version 5.03 of the Linkage package of computer programs (Lathrop & Lalouel, 1984), with the frequency of the gene fixed at 0.0001. For the likelihood calculations under genomic imprinting the penetrance in children of female gene carriers was assumed to be 0.0, irrespective of age.

### Results

One member (V–25) underwent clinical examination but did not have an MRI examination because of claustrophobia. In this patient computed tomography of the head and neck was performed instead. Individual IV–15, who is an obligate carrier and may well have the disease subclinically, and three of his five children who are at high risk of being affected, refused MR or CT screening despite several requests. All other participants completed the study.

*Glomus tumours*

Table I lists the clinical, hormonal and MR findings of the affected family members with one or more tumours. Examination of medical records revealed two deceased members (III-3, IV-18) in whom glomus tumours had been diagnosed by means of angiography and surgery. In the family member IV-4 who died from amyotrophic lateral sclerosis, no evidence of glomus tumours had been found on previously performed contrast enhanced CT examinations. The past medical and familial history of relatives II–1, II–2, III–1, III–2 and IV–5 was not suggestive of the presence of paragangliomas.

Clinical examination of the eight previously diagnosed glomus tumour patients revealed no new tumours. Among their relatives one (IV–10) complained of unsteadiness which she attributed to old age, but no tumours were found on physical examination. In another relative (IV–20) who also suffered from neurofibromatosis, bilateral carotid masses were felt in the neck.

In the eight known patients, the MR examinations of the head and neck demonstrated all previously diagnosed tumours and in addition, two hitherto unrecognised glomus tumours in two of the subjects (IV–17, V–13). In 6 undiagnosed relatives (IV–10, IV–13, IV–20, IV–22, V–64, V–66) 10 chemodectomas were found on MRI. In total MRI demonstrated 20 glomus tumours comprising eight carotid body tumours, three vagal body tumours and nine jugulotympanic tumours. Of the 14 living patients seven had multicentric lesions (50%). Tumour diameter ranged from 5 mm to 70 mm. A small vagal body tumour and a carotid body tumour were not visible on CT, but were confirmed by angiography.

In the patient with neurofibromatosis on MRI subcutaneous neurofibromas and a large skull lesion were found. The latter proved to be a so-called 'lambdoid defect' on skull roentgenograms (Resnick, 1989). Furthermore, in one relative, MRI showed a small cerebellar vascular malformation that was considered to be a coincidental finding.

*Functioning paragangliomas*

All participants underwent the urinary screening tests. Only one of the known patients (V–19) had a history that was indicative of a functioning paraganglioma, i.e. hypertension, episodic headaches, palpitations and heavy perspiration. This patient and two of the relatives (V–12, V–64) were found to have elevated urinary excretion of catecholamines. In two subjects (V–19, V–64) MRI and MIBG scintigraphy re-

**Table I** Demographic, clinical and imaging findings of paraganglioma patients

| *Pedigree identification* | *Age at diag. Gender* | *Signs and symptoms* | *Endocrin. findings* | *MRI findings* | *Diagnosis* | *Specials remarks* |
|---|---|---|---|---|---|---|
| A (III-3) | 49<br>M | Puls. Tinitus<br>Hearing loss | N.A. | N.A. | GJT Bilat. | Angiography<br>and Surgery |
| B (IV-10)[a] | 70<br>F | Vertigo<br>Unsteadiness | Hyper-<br>tension | GJT | GJT | |
| C (IV-11) | 53<br>F | Hearing loss | - | GJT | GJT | |
| D (IV-13)[a] | 64<br>M | None | - | GJT<br>GJT | GJT Bilat. | |
| E (IV-16) | 48<br>M | Hearing loss | - | GJT | GJT | |
| F (IV-17) | 45<br>M | Puls. Tinitus<br>Cervical mass | - | GVT<br>GCT | GVT[a]<br>GCT | |
| G (IV-18) | 19<br>F | N.A. | N.A. | N.A. | GJT | Died abroad from intra-<br>cranial extension GJT |
| H (IV-20)[a] | 48<br>M | Cervical mass<br>Bilateral | - | GCT<br>GCT | GCT Bilat.<br>Neurofibr. | Cutaneous neurofibromas and<br>Lambda skull defect on MRI. |
| I (IV-22)[a] | 43<br>M | None | ? | GCT<br>GJT | GCT<br>GJT | |
| J (V-11) | 29<br>M | Vagal nerve lesion<br>Middle ear mass | - | GJT | GJT | |
| K (V-12) | 27<br>M | Cervical mass | Catechol. | GCT | Function.<br>GCT | MIBG uptake by GCT |
| L (V-13) | 25<br>F | Hearing loss | - | GJT<br>GVT | GJT<br>GVT[a] | GVT not visible on CT,<br>confirmed by angiography |
| M (V-19) | 26<br>M | Facial nerve lesion<br>Middle ear mass | Hypert<br>Catechol. | GJT<br>Pheo | GJT<br>Pheo[a] | MIBG uptake by<br>left adrenal |
| N (V-64)[a] | 31<br>M | None | Catechol.<br>Hypert. | GVT/GCT<br>Pheo | GVT/GCT<br>Pheo | MIBG uptake by right adrenal<br>GCT only visible on angiogram |
| O (V-66)[a] | 27<br>M | None | - | GCT | GCT | |
| P (V-67) | 18<br>F | Cervical mass | - | GCT | GCT | |

[a]New patient or tumour diagnosis.

vealed an adrenal pheochromocytoma. After removal of the pheochromocytomas catecholamine production normalised in both patients. In the third person (V-12) there was a strong MIBG uptake by a small carotid body tumour. No other paragangliomas were present in this patient. On removal of the carotid body tumour, catecholamine production returned to normal. None of the others had signs or symptoms suggesting a hormonally active tumour.

### *Hereditary aspects*

The pedigree consisting of 99 family members older than 18 years showed a total of 16 patients with glomus tumours (Figure 1). Pheochromocytomas were found twice in combination with glomus tumours and did not occur separately. Eleven men and five women were affected. In six previously undiagnosed individuals the disease could be established by means of MRI. Nine male gene carriers had 16 affected and 18 unaffected children. In the offspring ($n = 15$) of three affected females no clinical evidence suggestive for paragangliomas or MRI abnormalities could be detected. In the 16 children of females who were at risk to be gene carrier no abnormalities were found either. Likelihood calculations were carried out to compare the regular autosomal dominant mode of inheritance with the alternative explanation of genomic imprinting. The results provide support for the hypothesis of genomic imprinting with odds as high as 23375:1.

### Discussion

The present study demonstrates that a considerable percentage of hormonally active and non-active paragangliomas are clinically occult both in known patients as well as in asymptomatic family members. By MR imaging and catecholamine testing we detected a number of new patients and new tumours that would otherwise only have been diagnosed in a much later stage or even not at all. It is therefore apparent that in the past, pedigrees of paraganglioma kindred groups have been incomplete.

The new cases of glomus tumour that were detected in this study occurred only in persons that had inherited the disease gene from their father. Our new findings are completely consistent with predictions based on the genomic imprinting theory, i.e. the gene is transmitted by both men and women, but only leads to disease manifestations when inherited via the father. Given a complete autosomal dominant transmission by males, the three affected females should have had seven or eight affected children, but they had none. These observations are summarised in our statistical evaluation, which provides convincing odds supporting the genomic imprinting hypothesis.

On the basis of this theory and the high percentage of asymptomatic patients found in our study, it can even be speculated that a proportion of ostensibly sporadic glomus tumours are in fact familial tumours that have been transmitted over several generations by the maternal line or by male patients with undetected tumours. A more accurate assessment of the proportion of isolated cases can only be obtained when the genetic defect has been characterised.

We dealt with a familial tumour syndrome. The common characteristics of the familial tumours are increased risk to the families of the patients, lower age of onset and multiplicity of tumours. The average age of tumour diagnosis in non-familial patients is 42.5 years, compared with a mean age at diagnosis of 38.8 years in our group (Grufferman *et al.*, 1980). In the fifth generation, in part due to our screening efforts, tumours were even diagnosed at a much earlier age (Table I). The more advanced age for tumour diagnosis in non-familial patients is probably due to the lack of suspicion and the initial absence of symptoms. Even in our group with highly aware individuals some affected members had reached an advanced age without any disturbing symptoms.

In the living patients, multiplicity (50%) and hormonal activity (21%) was diagnosed in a considerably higher frequency than has been reported in the past (Grufferman *et al.*,

**Figure 1** Pedigree of investigated family: MR-normal MRI; MRna MRI not available; glomus tumour patient; obligate gene carrier; possible gene carrier (female): functioning paraganglioma or pheochromocytoma; neurofibromatosis; deceased family member.

1980; Dunn *et al.*, 1986). This difference may entirely be accounted for by the high sensitivity of MRI and consistent testing for free urinary catecholamines with the highly accurate HPLC technique Stein & Black, 1991). These procedures were not used until recently.

As a screening method for paragangliomas, MRI proves to be far superior to physical examination. Small glomus tumours, in particular vagal and jugulo-tympanic tumours escape clinical recognition altogether.

MRI offers several advantages compared with other diagnostic possibilities such as angiography, contrast enhanced computed tomography and [$^{123}$I]MIBG scintigraphy that we have discussed in a previous report (van Gils *et al.*, 1991). Moreover, MRI can detect chemodectomas smaller than 5 mm, while contrast enhanced CT only allows detection of tumours greater than 8 mm (Vogl *et al.*, 1989). In our group this higher sensitivity of MRI was nicely illustrated by two tumours which, because of their small size, could only be confirmed by angiography and not by contrast enhanced CT. Sequential MR examinations can provide insight into the natural course of paragangliomas and provide essential information for genetic studies by exclusion of the disease in unaffected persons or by detection of tumours in a very early stage. A systematic search is currently under way to localise the specific genetic defect in hereditary glomus tumours. MR screening facilitates linkage analysis by increasing the number of affected members.

At present there are no guide-lines for screening at-risk relatives. Accurate risk assessment in family members depends on assumptions about the mode of inheritance and the role of genomic imprinting. Risk estimates for offspring of females will be very much different if one takes into account the possibility of genomic imprinting. When the gene responsible for familial chemodectomas has been mapped, further confirmation of the genomic imprinting theory will be possible and accurate risk calculations will indicate family members with a very high risk of being gene carriers. Regular MRI screening can be offered to those high risk subjects. The absence of adverse side effects will be of major advantage as it is necessary to follow these individuals for several years given the metachronous tumour occurrence (Grimley, 1989). Considering the fact that tumours are already found in patients aged 18 to 25 and that morbidity and mortality are directly related to size and extent of the tumour, it would seem wise to initiate screening around the age of 20. Urine analysis for catecholamines should always be included as clinical symptoms suggestive for functioning tumours may be sparse (van Gils *et al.*, 1990). This paucity of symptoms and the presence of only minimal biochemical abnormalities bears a striking resemblance to the behaviour of pheochromocytomas in MEN II (Stein & Black, 1991).

In the case of multiple paragangliomas and elevated catecholamine levels, such as our cases V-19, and V-64, one is not certain which tumour is active. In these cases MIBG scintigraphy is invaluable to localise the functioning lesion that has to be removed first (van Gils *et al.*, 1990). One patient showed cutaneous neurofibromas and a lambda defect in the skull together with two carotid body tumours. The association of neurofibromatosis with adrenal pheochromocytomas is well known but the combination of neurofibromatosis and multicentric extra-adrenal paragangliomas has only been reported once in English literature (DeAngelis *et al.*, 1987). A third case was found in German literature (Löblich & Baumann, 1960). Although rare this combination constitutes further evidence for Bolande's theory that both disorders are neurocristopathies (Bolande, 1974).

In conclusion, by means of prospective screening with MRI, this study provides further evidence for the theory that inheritance of familial paragangliomas is subject to genomic imprinting. When a paraganglioma is detected an extensive search should be performed for additional tumours and familial occurrence should be considered. For this purpose, screening with MRI is the method of choice, not only because it has no adverse side-effects, but also because of its very high sensitivity. In keeping with our results in another non-familial patient group hormonal activity was not an infrequent finding in this patient group. Because symptoms and signs are usually minimal, periodic examination of individuals at risk will allow the detection of tumours and hormonal activity in a presymptomatic and still localised stage. This in turn allows therapeutic intervention at an early stage thus providing a basis for secondary prevention.

The authors thank Mr R. Byrne and Ms P. Roes for technical assistance and Mr J. Beentjes for producing Figure 1.

### References

BOLANDE, R.P. (1974). The neurocristopathies: a unifying concept of disease arising in neural crest maldevelopment. *Hum Pathol.*, **5**, 409–429.

DEANGELIS, L.M., KELLEHER, M.B., POST, K.D. & FETELL, M.R. (1987). Multiple paragangliomas in neurofibromatosis; a new neuroendocrine neoplasia. *Neurology*, **37**, 129–133.

DUNN, G.D., BROWN, M.J., SAPSFORD, R.N., MANSFIELD, A.O., HEMINGWAY, A.P.,SEVER, P.S. & ALLISON, D.J. (1986). Functioning middle mediastinal paraganglioma (phaeochromocytoma) associated with intercarotid paraganglioma. *Lancet*, **i**, 1061–1064.

GLENNER, G.C. & GRIMLEY, P.M. (1974). Tumors of the extra-adrenal paraganglion system (including chemoreceptors). *Atlas of Tumor Pathology*: second series: fascicle 9. Armed Forces Institute of Pathology: Washington DC.

GRIMLEY, P.M. (1989). Multicentric paragangliomas and associated neuroendocrine tumors. In *Local Invasion and Spread of Cancer*, Brunson, K.W. (ed.) pp. 49–61. Kluwer Academic Publishers: Dordrecht.

GRUFFERMAN, S., GILLMAN, M.W., PASTERNAK, L.R., PETERSON, C.L. & YOUNG, W.G. (1980). Familial carotid body tumors: case report and epidemiologic review. *Cancer*, **46**, 2116–2122.

HALL, J.G. (1990). Genomic imprinting; review and relevance to human disease. *Am. J. Hum. Genet.*, **46**, 857–874.

LATHROP, G.M. & LALOUEL, J.M. (1984). Easy calculations of lod scores and genetic risks on small computers. *Am. J. Hum. Genet.*, **36**, 460–465.

LÖBLICH, H.J. & BAUMANN, A. (1960). Die verschiedenen Kombinationen geschwulstartiger Hyperplasien des Nervensystems. Ein Beitrag zur Pathogenese der Systemhyperplasien am Beispiel eines Sektionsfalles. *Zentbl. Allg. Pathol.*, **101**, 159–165.

PARKIN, J.L. (1981). Familial multiple glomus tumors and pheochromocytomas. *Ann Otol.*, **90**, 60–63.

RESNICK, D. (1989). Bone and Joint Imaging, pp. 1220–1221. W.B. Saunders: Philadelphia.

STEIN, P.P. & BLACK, H.R. (1991). A simplified diagnostic approach to pheochromocytoma. A review of the literature and report of one institution's experience. *Medicine*, **70**, 46–66.

VAN DER MEY, A.G.L., MAASWINKEL-MOOY, P.D., CORNELISSE, C.J., SCHMIDT, P.J. & VAN DE KAMP, J.J.P. (1989). Genomic imprinting in hereditary paragangliomas; evidence for a new genetic theory. *Lancet*, **ii**, 1291–1294.

VAN GILS, A.P.G., VAN DER MEY, A.G.L., HOOGMA, R.P.J.M., FALKE, T.H.M., MOOLENAAR, A.J., PAUWELS, E.K.J. & VAN KROONENBURGH, M.J.P.G. (1990). I-123 Metaiodobenzylguanidine in the detection of chemodectomas in the head and neck region. *J. Nucl. Med.*, **31**, 1147–1155.

VAN GILS, A.P.G., FALKE, T.H.M., VAN ERKEL, A.R., ARNDT, J.W., SANDLER, M.P., VAN DER MEY, A.G.L. & HOOGMA, R.P.L.M. (1991). MRI and MIBG imaging of pheochromocytomas and extra-adrenal functioning paragangliomas. *Radiographics*, **11**, 37–57.

VOGL, T., BRÜNING, R., SCHEDEL, H., KANG, K., GREVERS, G., HAHN, D. & LISSNER, J. (1989). Paragangliomas of the jugular bulb and carotid body: MR Imaging with short sequences and Gd-DTPA enhancement. *AJR*, **153**, 583–587.